# Lumbar hernia associated with chronic obstructive pulmonary disease (COPD)

**Published:** 2013

**Authors:** Tao Xu, Shuwei Zhang, Huaying Wang, Wanjun Yu

**Affiliations:** 1 Tao Xu, MD, Department of Respiration, The People’s Hospital of Yinzhou, The Affiliated Yinzhou Hospital, Ningbo University, 251 Baizhang East Road, Ningbo, Zhejiang, 315040, The People’s Republic of China.; 2 Shuwei Zhang, MD, Department of Urological Surgery, The No.2 Hospital of Yinzhou, 1 Qianhe Road, Ningbo, Zhejiang, 315040, The People’s Republic of China.; 3 Huaying Wang, MD, PhD, Department of Respiration, The People’s Hospital of Yinzhou, The Affiliated Yinzhou Hospital, Ningbo University, 251 Baizhang East Road, Ningbo, Zhejiang, 315040, The People’s Republic of China.; 4 Wanjun Yu, MD, PhD, Department of Respiration, The People’s Hospital of Yinzhou, The Affiliated Yinzhou Hospital, Ningbo University, 251 Baizhang East Road, Ningbo, Zhejiang, 315040, The People’s Republic of China.

**Keywords:** Lumbar hernia, Chronic obstructive pulmonary disease

## Abstract

Lumbar hernias are very rare posterolateral abdominal wall hernias, and they are spontaneous in most adult patients. Here we report two cases of spontaneous lumbar hernias associated with chronic obstructive pulmonary disease (COPD). Some factors such as chronic cough, poor nutritional status and old age in patients with COPD would contribute to lumbar hernia.

## INTRODUCTION

Lumbar hernias are very rare posterolateral abdominal wall hernias which are located in the thoracolumbar region. Until now, there have been fewer than 300 cases reported in the world literature.^1^ Aetiologically, lumbar hernias can be divided into congenital, primary or acquired sets secondary to trauma or surgery. The majority of patients either are asymptomatic or complain of a posterior lump, flank discomfort or chronic backache. Some factors including raised intra-abdominal pressure and muscle atrophy are believed to predispose to primary lumbar hernias.^2^ Here we present two cases of primary lumbar hernias associated with chronic obstructive pulmonary disease (COPD) to detect some relationship between these two diseases.

***Case-1*** **:**A 67-year-old male with repeated cough and shortness of breath for seven years presented to our pulmonary center. He was diagnosed with chronic bronchitis and treated with intermittent oral cefaclor and salbutamol in the local clinic in the past three years. The patient was a truck farmer and had a history of smoking for forty years.

**Fig.1:**
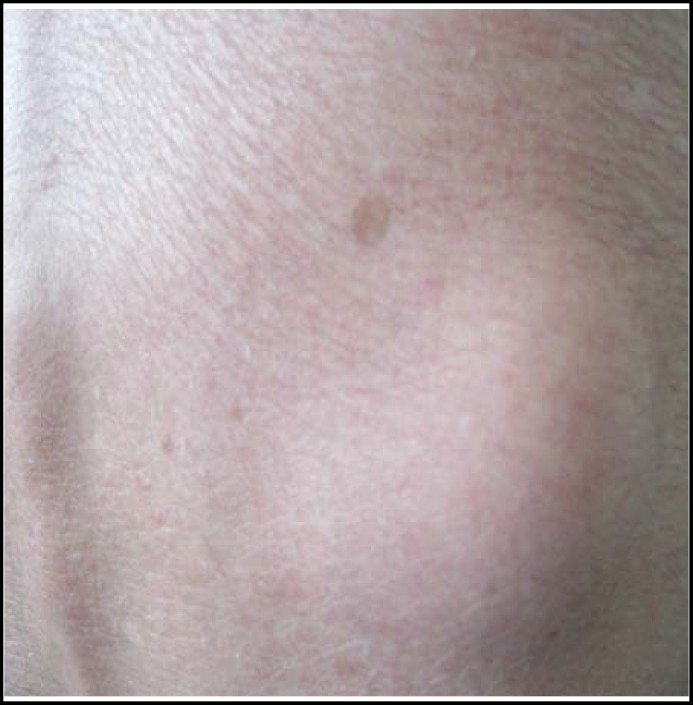
Protrusion in the lateral right the lumbar area

There was no previous history of surgery or injury. Due to lack of response from prescribed medications, he was referred to our center for further investigations. On physical examination, the patient’s chest was symmetrical and barrel shaped, with diminished breath sounds heard. Pulmonary function tests showed forced vital capacity (FVC) and forced expiratory volume in 1s (FEV1) was below lower limit of normal (FEV1/FVC was 60% and FEV1 was 55%). Chest CT scan showed significant emphysematous changes in his lung. The diagnosis of COPD was established. On abdominal examination, there was a 6x4cm soft, non-tender, smooth-surface swelling in the right lumbar region. (Fig.1). The mass could disappear when lying down. He recalled its presentation five years before. Abdomen Computed tomography (CT) scan confirmed the presence of right lumbar hernia containing fatty tissue. After treatment of regular oral theophylline sustained release tablets and prednisone  acetate tablets, his respiratory symptoms improved obviously. The patient refused operation and was discharged from the hospital one week later.

**Fig.2:**
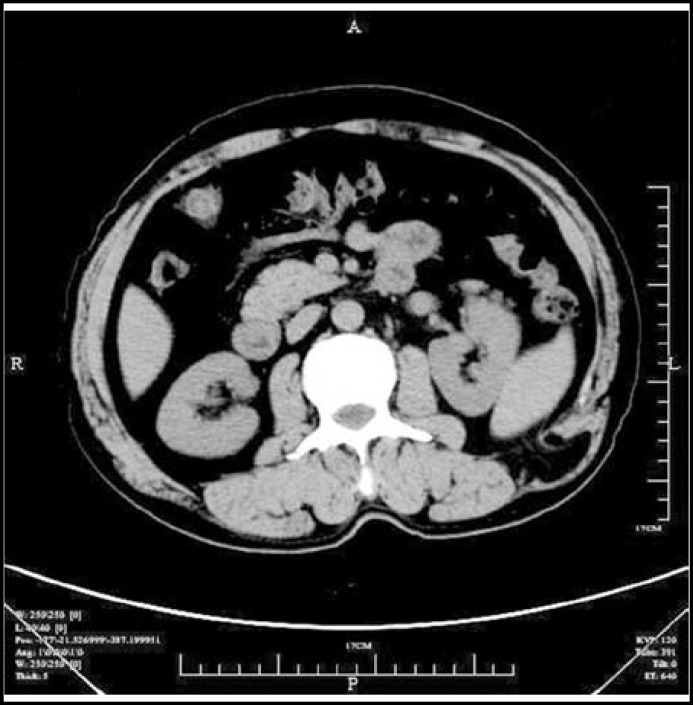
Computed tomography (CT) scan demonstrating left lumbar hernia containing fatty tissue

***Case – 2:*** The patient was a 61- year- old male, presenting with gradually increasing painless and reducible left flank mass for the past two years , admitted to our hospital for operation. He was a non-smoker and had a medical history of COPD. He had been working as a cargador for 20 years. In the past ten years, He had always been coughing and mild breathless intermittently, especially in winter and spring .There was no history of trauma, surgery. On admission, his pulmonary function tests revealed FEV1/FVC was 63% and FEV1 was 70%. On abdominal examination, there was a 6x5cm soft, non-tender, smooth-surface and reducible swelling in the left lumbar region. Computed tomography (CT) scan of the abdomen was carried out which confirmed the presence of left lumbar hernia containing fatty tissue (Fig.2). The patient was placed in a left lateral position of routine primary repair. The result of his surgical repair was good with no recurrence in the follow-up of six months.

## DISCUSSION

Lumbar hernia is an abnormal protrusion in the flank. Since the original description by de Garangeor in 1731, hundreds of cases have been described.^3^ Lumbar hernia occurs in weak anatomic points or other portions of the posterior abdominal wall. The superior and inferior lumbar triangles are the most common sites where it herniates.

Lumbar hernia can be congenital or acquired. Congenital lumbar hernia is extremely rare and presents in children, due to defect of the lumbar wall. Acquired hernia can be classified spontaneous or secondary following trauma, surgery or inflammation. In most adult patients, lumbar hernias are still spontaneous, which is due to raised intra-abdominal pressure and acquired predisposition such as muscle atrophy, increasing age, extremes of weight and chronic debilitating disease.^4^ Neither of our cases has history of surgical procedure, infection, or trauma. The lumbar hernias are believed to be spontaneous. Additionally, both of two cases are diagnosed with COPD, which may suggest some links between the two diseases.

COPD is now seen as a systemic disease affecting multiple organs and systems and malnutrition is present in many moderate or severe cases.^5^ It has become a major public health problem worldwide nowadays. The incidence has been increasing sharply recently in China, due to huge smokers, expanding aging population and severe air pollution. People with COPD can spend more energy on breathing than healthy individuals and multiple factors such as decreased food intake, high systemic inflammatory response can lead to malnutrition.^6^ Changes in nutritional status are a very common complication in these patients. Our patients are elderly male and manual laborers. Heavy manual labor, malnutrition and increasing age must make muscle atrophied. Repeatedly chronic cough may lead to raising intra-abdominal pressure. All these would contribute to lumbar hernia. It hints at some links between lumbar hernia and COPD. More aspects of the relationship should await further investigation, because of few cases. Usually, the diagnosis of lumbar hernia is not difficult and based on history and physical examination. CT scan or MRI is helpful for co-diagnosis.

Although rarely resulting in strangulation, lumbar hernia often recommends surgical repair at the time of discovery.^7^ Surgery for lumbar hernia is simple and safe with few complications, especially through laparoscopic procedures. In spite of COPD, our patient’s conditions tolerated surgery, but one patient refused surgery. We performed the open approach for the patient who wanted surgery successfully, and explained the risks of strangulation to the patient who refused surgery.

## CONCLUSION

Lumbar hernia is a rare disease. Our two patients of lumbar hernia were associated with chronic obstructive pulmonary disease (COPD), chronic cough, poor nutritional status and old age could be the risk factors. More studies should be performed to make it clear.

## Authors Contribution

TX: Conceived, designed and did editing of manuscript.

SZ and HW: Did data collection and manuscript writing.

WY: Did review and final approval of manuscript.
